# Blood-based tumor mutational burden as a biomarker in unresectable non-small cell lung cancer treated with chemoradiotherapy and durvalumab

**DOI:** 10.3389/fonc.2025.1681420

**Published:** 2025-10-22

**Authors:** Henrik Horndalsveen, Vilde Drageset Haakensen, Tesfaye Madebo, Bjørn Henning Grønberg, Tarje Onsøien Halvorsen, Jussi Koivunen, Kersti Oselin, Saulius Cicenas, Nina Helbekkmo, Marianne Aanerud, Jarkko Ahvonen, Maria Silvoniemi, Maria Moksnes Bjaanæs, Saima Farooqi, Daniel Nebdal, Astrid Marie Dalsgaard, Britina Kjuul Danielsen, Mari Børve, Tonje Sofie Dalen, Åsa Kristina Öjlert, Åslaug Helland

**Affiliations:** ^1^ Institute for Cancer Research, Department of Cancer Genetics, Oslo University Hospital, Oslo, Norway; ^2^ Department of Oncology, Oslo University Hospital, Oslo, Norway; ^3^ Department of Clinical Medicine, University of Oslo, Oslo, Norway; ^4^ Department of Pulmonology, Stavanger University Hospital, Stavanger, Norway; ^5^ Department of Clinical Science, University of Bergen, Bergen, Norway; ^6^ Department of Clinical and Molecular Medicine, NTNU, Norwegian University of Science and Technology (NTNU), Trondheim, Norway; ^7^ Department of Oncology, St. Olavs Hospital, Trondheim University Hospital, Trondheim, Norway; ^8^ Department of Oncology and Radiotherapy, Oulu University Hospital, Oulu, Finland; ^9^ Cancer Center, Medical Research Center Oulu, Oulu University Hospital, Oulu, Finland; ^10^ Oncology and Haematology Clinic, North Estonia Medical Centre, Tallinn, Estonia; ^11^ Department of Thoracic Surgery and Oncology, National Cancer Center, Affiliate of Vilnius University Hospital Santaros Klinikos, Vilnius, Lithuania; ^12^ Department of Pulmonology, University Hospital of North Norway, Tromsø, Norway; ^13^ Department of Thoracic Medicine, Haukeland University Hospital, Bergen, Norway; ^14^ Tays Cancer Center, Department of Oncology, Tampere University Hospital, Tampere, Finland; ^15^ Department of Pulmonary Medicine, Turku University Hospital, Turku, Finland

**Keywords:** locally advanced NSCLC, immunotherapy, biomarker, TMB, circulating tumorDNA

## Abstract

**Introduction:**

Chemoradiotherapy followed by durvalumab is a potentially curative treatment for unresectable, locally advanced non-small cell lung cancer (NSCLC), but clinical outcomes remain highly variable. Identifying robust biomarkers is essential to refine treatment selection and enable risk-adapted strategies.

**Methods:**

In this multicenter, prospective cohort study, 86 patients with unresectable stage III NSCLC were treated with chemoradiotherapy followed by durvalumab. Baseline plasma samples underwent genomic profiling and blood tumor mutational burden (bTMB) assessment using targeted next-generation sequencing. Associations between bTMB, circulating tumor DNA (ctDNA) alterations, PD-L1 expression, and progression-free survival (PFS) were evaluated using a one-sided significance threshold of *p* < 0.10.

**Results:**

Median PFS was 18.9 months (95% CI: 14.7–not reached), and median bTMB was 6.6 mutations/megabase. In univariable analysis, high bTMB was associated with longer PFS using both the prespecified 8.5 mut/Mb cut-off (HR: 0.65; *p* = 0.088) and the median 6.6 mut/Mb cut-off (HR: 0.52; *p* = 0.016). PD-L1 ≥ 1% was associated with longer PFS (HR: 0.38; *p* = 0.0003), while *STK11*, *KEAP1*, or *NFE2L2* mutations in ctDNA were linked to shorter PFS (HR: 1.84; *p* = 0.040). In multivariable analysis, PD-L1 remained significantly associated with PFS in both models, while bTMB and *STK11*/*KEAP1*/*NFE2L2* mutations were significant using the 6.6 mut/Mb cut-off.

**Conclusion:**

High bTMB, PD-L1 expression ≥ 1%, and absence of *STK11*/*KEAP1*/*NFE2L2* mutations were associated with longer PFS. These findings support integrating multiple biomarkers to improve risk stratification and personalize treatment in unresectable stage III NSCLC.

**Clinical Trial Registration:**

The study is registered on www.clinicaltrials.gov (ClinicalTrials.gov identifier: NCT04392505).

## 1 Introduction

Approximately 20-30% of non-small cell lung cancer (NSCLC) patients are diagnosed with stage III disease (1, 2). Patients with unresectable stage III disease and a good performance status may undergo radical therapy using radiotherapy (60–66 Gy) with concurrent platinum-based doublet chemotherapy (3). The PACIFIC trial, supported by real-world data, demonstrated superior outcomes when chemoradiotherapy (CRT) is consolidated with one year of the anti-programmed death-ligand 1 (PD-L1) inhibitor durvalumab (4–6). However, many patients relapse despite durvalumab treatment, while up to 20% of patients not receiving durvalumab achieve long-term disease-free survival. These observations highlight the need for new biomarkers to better predict treatment responses and enable personalized, risk-adaptive treatment.

Currently, PD-L1 expression is the most clinically useful yet imperfect biomarker for predicting the efficacy of immune checkpoint inhibitors (ICIs) in NSCLC (7). Tumor mutational burden (TMB), defined as the number of somatic non-synonymous mutations per coding area of the tumor genome, has emerged as another promising biomarker (8, 9). Theoretically, a high TMB may increase tumor neoantigen formation to enhance neoantigen-specific T-cell responses and improve sensitivity to ICIs (10–12). While TMB was originally analyzed in tumor tissue samples by whole exome sequencing (WES), mounting evidence suggests that targeted gene panels may offer comparable precision, provided that the panel size is sufficient (≥1 Mb) (8, 13). Recently, methods for determining TMB in circulating tumor DNA (ctDNA) have emerged. Blood-based TMB (bTMB) analysis involves less invasive sampling and is the only viable option in cases where tumor tissue is difficult to obtain. Furthermore, bTMB may be less susceptible to tumor heterogeneity and allows for repeated assessments during treatment (13). However, the consistency between liquid- and tissue-based TMB analyses and the optimal approach remain to be defined (8, 11).

In advanced NSCLC, studies have demonstrated that high TMB correlates with greater benefit from ICIs, particularly when immunotherapy is administered alone and not in combination with chemotherapy (14–23). Further, TMB in this context appears to be independent of PD-L1 expression (7, 8). In early-stage resected NSCLC, high tissue TMB (tTMB) has been reported to predict improved locoregional control after post-operative radiotherapy, suggesting that TMB may serve as a biomarker of radiosensitivity (24). Still, the role of TMB in locally advanced NSCLC treated with chemoradiation and durvalumab remains underexplored (25–28).

Detection of specific mutations offers an alternative approach to examine the mutational landscape of NSCLC for prognostic and predictive biomarkers. *STK11* mutations impair DNA damage repair, while *KEAP1*/*NFE2L2* mutations enhance the ability of cancer cells to tolerate oxidative stress, both contributing to radiotherapy resistance (24, 29). Additionally, alterations in *STK11*, *KEAP1*, and *NFE2L2* are linked to immunologically cold tumor microenvironments, potentially serving as negative predictive biomarkers for immunotherapy (29, 30).

The Durvalumab After ChemoRadiotherapy (DART) study enrolled patients with unresectable stage III NSCLC eligible for CRT followed by durvalumab. The aim was to explore the biology underlying treatment response and resistance. Here, we evaluate TMB, PD-L1 expression, and ctDNA-based pathogenic gene alterations as biomarkers in this setting, with a primary focus on associations between bTMB and progression-free survival (PFS).

## 2 Materials and methods

### 2.1 Patients, study design and treatment

The DART study is a multicenter phase II translational and biomarker study conducted at ten hospitals in Norway, Finland, Lithuania, and Estonia. Patients with unresectable stage III NSCLC were enrolled and treated with curatively intended CRT, consisting of two cycles of platinum-based doublet chemotherapy every three weeks and radiotherapy at 2 Gy per fraction to a total dose of 60–66 Gy. Patients without disease progression following CRT received durvalumab 1500 mg every four weeks, preferably starting within five weeks of CRT completion, and continued until progression, intolerable toxicity, or a maximum duration of 12 months. Participants not starting durvalumab were excluded from the analyses.

### 2.2 Ethics statement

The study adhered to the Declaration of Helsinki (31), Good Clinical Practice, and all applicable laws and institutional guidelines. Approval was granted by the Regional Committee for Medical and Health Research Ethics (reference 48665, November 28, 2019). All participants provided informed consent. The trial is registered at ClinicalTrials.gov (NCT04392505).

### 2.3 Clinical assessments

Baseline imaging included CT of the chest/upper abdomen, MRI or CT of the brain, and whole body 18F-FDG PET/CT. Tumor evaluation by CT was performed between completion of CRT and the first durvalumab infusion, every 12 weeks during durvalumab therapy, and for the next two years, then every 26 weeks for an additional three years until progression or death. Supplemental MRI and PET/CT were conducted if clinically indicated. Tumor response was assessed per Response Evaluation Criteria in Solid Tumors (RECIST) version 1.1 (32). Lesions receiving radiotherapy as part of CRT were considered measurable, since radiotherapy was part of the study protocol and applied to all baseline lesions. Disease progression required radiologic progression by RECIST 1.1, supported by one of the following: 1) a biopsy or PET showing clear progression, 2) clinical deterioration, or 3) a confirmatory CT scan performed at least four weeks after the initial scan. If progression was confirmed, the date of the first scan indicating progression was recorded. The primary endpoint was PFS, defined as time from the start of durvalumab to disease progression or death from any cause. Overall survival (OS) was calculated from durvalumab initiation to death.

### 2.4 Tumor tissue collection, sequencing and tTMB calculation

Formalin-fixed paraffin-embedded (FFPE) tumor tissue and matched buffy coat samples for germline variant filtering were obtained at baseline. HE-stained FFPE sections were reviewed by a pathologist to confirm tumor content. DNA was extracted using the AllPrep DNA/RNA FFPE Kit (Qiagen) for tumor and QIAamp DNA Blood Mini Kit (Qiagen) for buffy coats. DNA concentration and quality were assessed using Qubit (ThermoFisher Scientific), Nanodrop (ThermoFisher Scientific), and Genomic DNA ScreenTape (Agilent). Samples with tumor DNA concentration > 3 ng/µl and matched buffy coats were submitted for sequencing. WES was performed at the OUH Genomics Core Facility using the Twist Biosciences Library Preparation Kit and the Twist Human Comprehensive Exome Enrichment Kit (Illumina). Sequencing (2 × 150 bp) was performed on a NovaSeq6000 system at average coverages of 150× (tumor) and 50× (buffy coat). Sequencing reads were aligned to the human reference genome GRCh38 using the Burrows–Wheeler Aligner (BWA_MEM2). Somatic variants were identified with GATK Mutect2 (v4.2.6.1) and Strelka (v2.9.10), and annotated using the Personal Cancer Genome Reporter (33). Variants with a variant allele frequency (VAF) ≥ 5% and tumor read depth ≥ 100× were included in tTMB calculation, defined as the number of non-synonymous SNVs and indels per megabase of targeted exome. Additional details are provided in the **Supplementary Methods**.

### 2.5 Plasma sample collection, sequencing and bTMB calculation

Peripheral blood was collected in three 10 ml cfDNA BCT tubes (Streck) at baseline. Plasma was separated via two-step centrifugation before storage at –80 °C. cfDNA was extracted from 8 ml plasma using the Mag-Bind cfDNA Kit (Omega Bio Tek) on an automated platform (Opentrons OT-2, KingFisher Flex). DNA library preparation followed established protocols (34) including dA-tailing, adaptor ligation, and indexing PCR, with intermediate quality control using the Agilent 4150 TapeStation. Target regions were captured by hybridization using TACS (target capture sequences). The NeoThetis Pan Cancer Plus assay (MEDICOVER Genetics), targeting 222 cancer-related genes and a total of 1.25 Mb, was used to identify single nucleotide variants (SNVs), small insertions and deletions (indels), copy number amplifications (CNAs) and structural rearrangements (**Supplementary Table 1**). Captured libraries were sequenced on a NovaSeq6000 platform (Illumina). Reads were demultiplexed using bcl-convert (v4.2), with poor-quality reads and adaptor sequences removed before alignment to GRCh37 using the Burrows-Wheeler algorithm (35). Duplicate reads were grouped by unique adaptor families to generate consensus reads. To further refine the set of positive variant calls, a statistical error correction model (at base-pair resolution), followed by a filtering bioinformatics pipeline, was applied. ctDNA variant calling was performed *de novo* (not tumor-guided) and variants were classified per AMP guidelines using automated tiering (VarSomeClinical), followed by manual curation by at least two variant analysts. Variants were excluded if they had VAF < 0.25%, population frequency > 1% (gnomAD), were synonymous, or were deemed low-confidence. For bTMB calculation, only SNVs and indels in targeted regions with ≥ 1000× coverage were counted. Additional details are provided in the **Supplementary Methods**.

### 2.6 Statistical analysis

PFS and OS were estimated using the Kaplan-Meier method. Follow-up time was calculated using reverse Kaplan-Meier. TMB was analyzed as a categorical variable (low vs. high) using several cut-offs, including 8.5 mut/Mb (protocol-prespecified primary) and the cohort median. The 8.5 mut/Mb cut-off was set when the protocol was planned in 2019, informed by metastatic NSCLC medians (7–10 mut/Mb) and the then-common use of 10 mut/Mb. Given limited data in stage III NSCLC and the expectation of slightly lower TMB, 8.5 mut/Mb was chosen to balance biological plausibility and statistical power. *STK11*, *KEAP1* and *NFE2L2* mutations were analyzed as a grouped variable, reflecting shared biology linked to treatment resistance and the low individual frequencies of these alterations. Associations between patient characteristics and genomic variables at baseline were assessed using Fisher’s exact test or Chi-square test for categorical variables. For categorical vs. continuous variables, Wilcoxon rank-sum (two groups) or Kruskal-Wallis (more than two groups) tests were applied. Correlations were examined using Spearman’s method. Associations between clinical/genomic characteristics and PFS were assessed using log-rank tests and Cox proportional hazards models. Key variables significantly associated with outcome in univariable analysis were further evaluated in multivariable Cox regression models, adjusted for age and performance status, as established prognostic factors. As prespecified in the study protocol, the significance threshold (alpha) was set at 0.10, with one-sided p-values to test effects in the expected direction. Statistical analyses were performed in R(v4.1.1)

## 3 Results

### 3.1 Clinical and treatment characteristics

**Figure 1:**
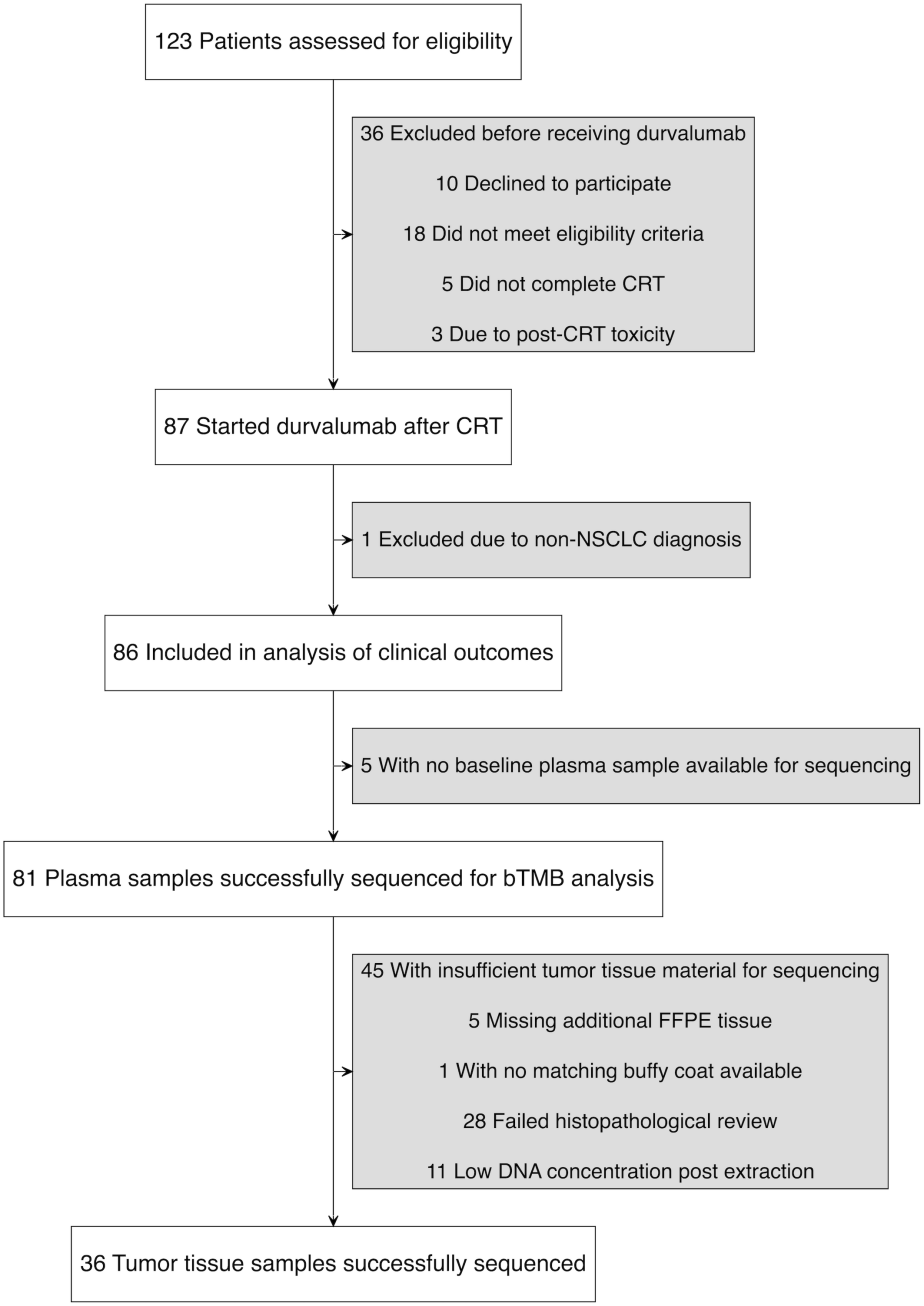
Study flow diagram illustrating patient enrollment, exclusions, and sample availability.

Between May 5, 2020, and September 7, 2023, 123 patients were screened, of whom 90 met all eligibility criteria and completed CRT. Of these, 87 initiated durvalumab (**Figure 1**). One patient was excluded after a re-examination of the lung tumor biopsy concluded that it was a metastasis from rectal cancer. Another patient was found to have stage IIB disease upon later radiological review but was included in the primary analysis since the patient had unresectable NSCLC and was treated according to protocol. Baseline clinical characteristics of the 86 patients are shown in **Table 1**. The median age was 69 years (range 36-85), 60% (n=52) were male and 95% (n=82) had a history of smoking. Histologically, 57% (n=49) of tumors were squamous cell carcinoma and 41% (n=35) had PD-L1 expression <1%. The median time from end of CRT to durvalumab initiation was 24 days (range 6-45) with a median of 11 durvalumab infusions administered (range 1–13).

**Table 1:** Patient characteristics.

Clinical characteristics	N = 86
Age: median, (range)	69, (36 – 85)
Sex	
Male	52 (60.5%)
Female	34 (39.5%)
Smoking	
Current	26 (30.2%)
Former	56 (65.1%)
Never	4 (4.7%)
Performance status	
0	34 (39.5%)
1	52 (60.5%)
Histology	
Adenocarcinoma	31 (36.0%)
Squamous cell carcinoma	49 (57.0%)
NSCLC NOS	6 (7.0%)
PD-L1 expression	
Negative (< 1%)	35 (40.7%)
Positive (≥ 1%)	51 (59.3%)
Stage (TNM 8th edition)	
IIB	1 (1.2%)
IIIA	38 (44.2%)
IIIB	39 (45.3%)
IIIC	8 (9.3%)

### 3.2 Genomic characteristics

**Figure 2:**
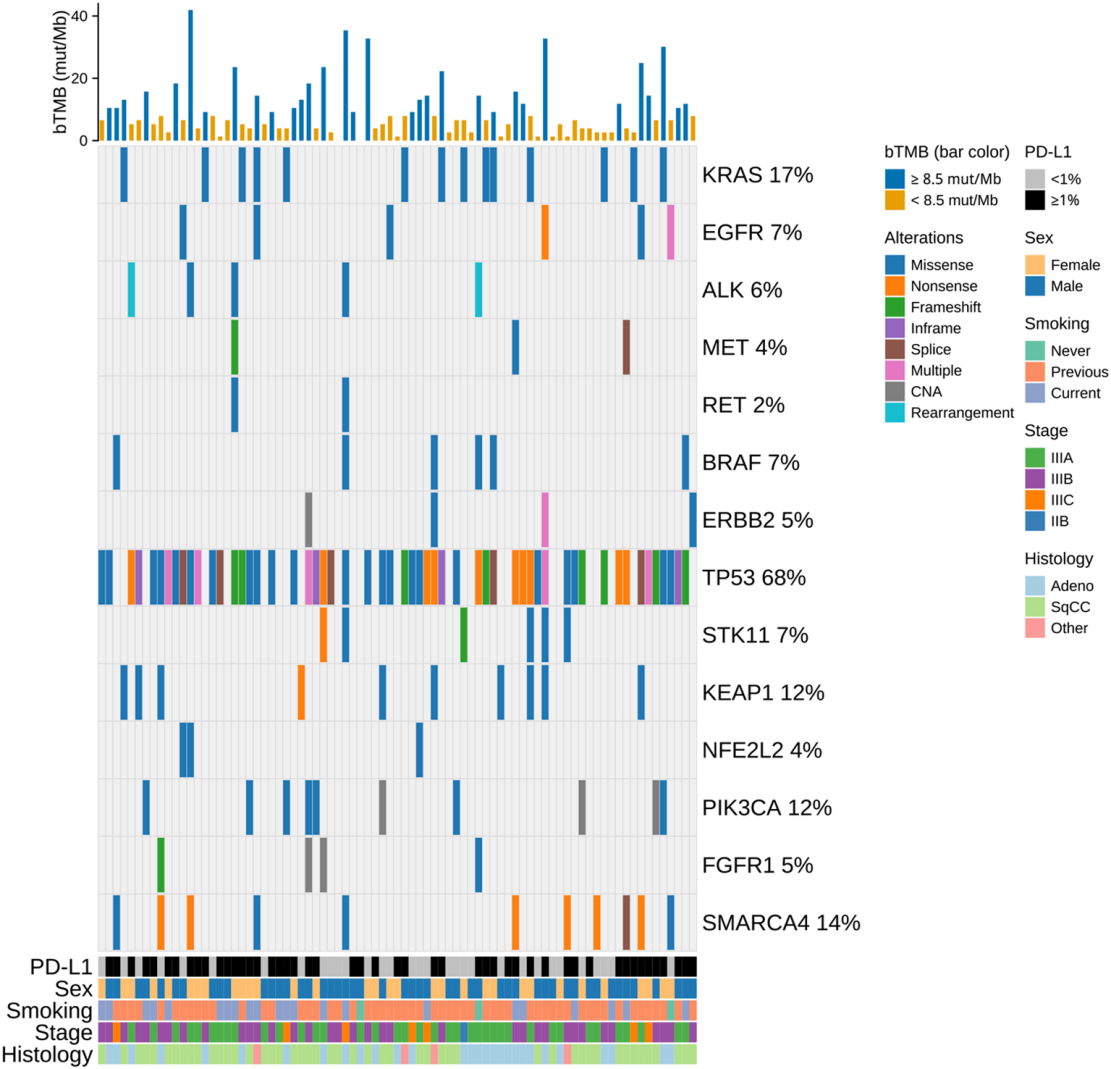
Oncoprint of the study population showing functionally relevant genomic alterations detected in plasma ctDNA. Clinical annotations (PD-L1, sex, smoking status, stage, histology) and bTMB are displayed as overlays; alteration types are color-coded, and each column represents one patient. Bars above indicate bTMB scores, color-coded by bTMB category using a cut-off of 8.5 mut/Mb. bTMB, blood tumor mutational burden; CAN, copy number amplification; mut/Mb, mutations per megabase; SqCC, squamous cell carcinoma.

Baseline plasma samples for sequencing were available from 81 of 86 patients, all of which passed quality control. The median bTMB was 6.6 mut/Mb (range: 0–41.9 mut/Mb, **Figure 2**). Using the prespecified cutoff of 8.5 mut/Mb, 49 patients were categorized as bTMB low, and 32 as bTMB high. No significant associations were found between bTMB and baseline patient characteristics. The oncoprint summarizes functionally relevant genomic alterations detected in plasma ctDNA (**Figure 2**). *TP53* was the most frequently altered gene in plasma (68% of patients) followed by KRAS (17%). Alterations in *STK11*, *KEAP1* or *NFE2L2* were observed in 21% of patients. Patients with *TP53* mutations in plasma ctDNA had higher bTMB (*p* < 0.001). No other individual mutations showed significant associations with bTMB, but combined *STK11/KEAP1/NFE2L2* alterations were linked to higher bTMB (*p* = 0.046) and PD-L1 negativity (*p* = 0.035).

Of the 81 patients with sequenced plasma samples, 36 had tumor tissue with sufficient tumor content for WES (**Figure 1**). The median tTMB was 11.6 mut/Mb (range: 2.6–49.5 mut/Mb). The correlation between bTMB and tTMB was moderate (Spearman’s ρ = 0.50, *p* = 0.002), with tTMB values being significantly higher (*p* = 0.012). When using the median to categorize TMB as high or low, 75% (27/36) of patients were concordantly classified by bTMB and tTMB. Neither bTMB nor tTMB significantly correlated with PD-L1 expression, although a weak trend toward higher bTMB was seen in patients with PD-L1 ≥ 1% (bTMB: Spearman’s ρ = 0.15, *p* = 0.172; tTMB: Spearman’s ρ = 0.01, *p* = 0.937; **Supplementary Figure 1**). Among patients with matched plasma and tissue samples (gene-level presence/absence comparison), 60% of mutations (SNVs and indels) in key genes were detected in both plasma and tumor tissue, while 25% were exclusive to tumor and 15% to plasma (**Supplementary Figure 2**).

### 3.3 Clinical outcomes

At the cut-off date (December 1, 2024), median follow-up was 33.1 months (IQR 22.3–35.6). The median PFS was 18.9 months (95% CI: 14.7–not reached, NR). A total of 47 patients (54.7%) had experienced a progression event at a median of 8.3 months. Of these, 20 had local recurrences, 19 had distant metastases, two had both local recurrence and distant metastasis, and six died without documented progression. The median OS was not reached. The 12- and 24-month OS rates were 87.2% (95% CI: 80.4–94.5) and 71.5% (95% CI: 62.1–82.2), respectively. In univariable analyses of baseline clinical characteristics and PFS, age ≥75 years (HR: 2.02; 95% CI: 0.80–5.13; p = 0.07) and male sex (HR: 1.63; 95% CI: 0.88–3.02; p = 0.06) were associated with shorter PFS (**Supplementary Figure 3**). No significant associations with PFS were found for smoking status, ECOG performance status, disease stage, histology, or time from CRT to durvalumab (<28 vs. ≥28 days).

### 3.4 Association between TMB and PFS

**Figure 3:**
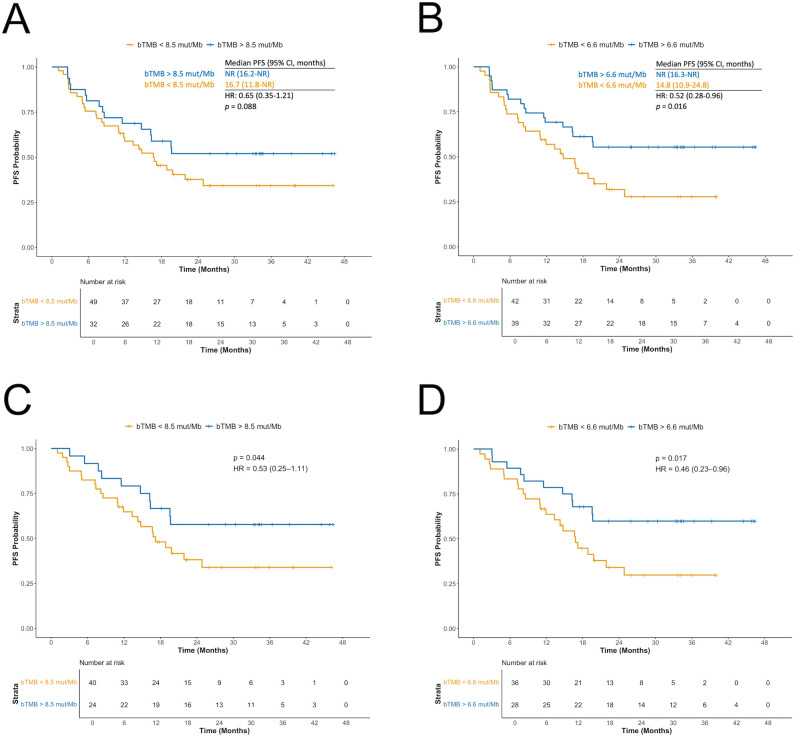
Association between blood tumor mutational burden (bTMB) and progression-free survival (PFS) based on two different cut-offs. **(A)** bTMB </> 8.5 mutations per megabase (mut/Mb) in the full cohort. **(B)** bTMB </> median value of 6.6 mut/Mb in the full cohort. **(C)** bTMB </> 8.5 mut/Mb after excluding patients with *STK11*, *KEAP1*, or *NFE2L2* mutations detected in blood. **(D)** bTMB </> 6.6 mut/Mb after excluding patients with *STK11*, *KEAP1*, or *NFE2L2* mutations detected in blood. bTMB, blood tumor mutational burden; mut/Mb, mutations per megabase; PFS, progression-free survival.

Using the prespecified cut-off of 8.5 mut/Mb, patients with high bTMB had improved PFS compared to those with low bTMB (HR: 0.65; 95% CI: 0.35–1.21; *p* = 0.088; **Figure 3A**). The median PFS was NR for high bTMB (95% CI: 16.2–NR) and 16.7 months for low bTMB (95% CI: 11.8–NR). Applying the median value of 6.6 mut/Mb as an alternative cut-off, high bTMB was significantly associated with longer PFS (HR: 0.52; 95% CI: 0.28–0.96; *p* = 0.016; **Figure 3B**). The median PFS was NR (95% CI: 16.3–NR) in high bTMB vs. 14.8 months (95% CI: 10.9–24.8) in low bTMB. Higher thresholds (10, 16, and 20 mut/Mb) did not yield significant associations with PFS (*p* = 0.181, *p* = 0.369 and *p* = 0.241, respectively; **Supplementary Figure 4**). Notably, very few patients were classified as having high bTMB when applying these higher thresholds.

Excluding patients with *STK11*/*KEAP1*/*NFE2L2* mutations strengthened the association between high bTMB and longer PFS for both the 8.5 mut/Mb cut-off (HR: 0.53; 95% CI: 0.25–1.11; *p* = 0.044; **Figure 3C**) and 6.6 mut/Mb (HR: 0.46; 95% CI: 0.23–0.96; *p* = 0.017; **Figure 3D**). The 10 mut/Mb cut-off also reached significance (HR: 0.59; 95% CI: 0.27–1.30; *p* = 0.096).

Among the 36 patients with tTMB data, no significant PFS difference was observed between high and low tTMB groups.

### 3.5 Association between PD-L1 expression and PFS

**Figure 4:**
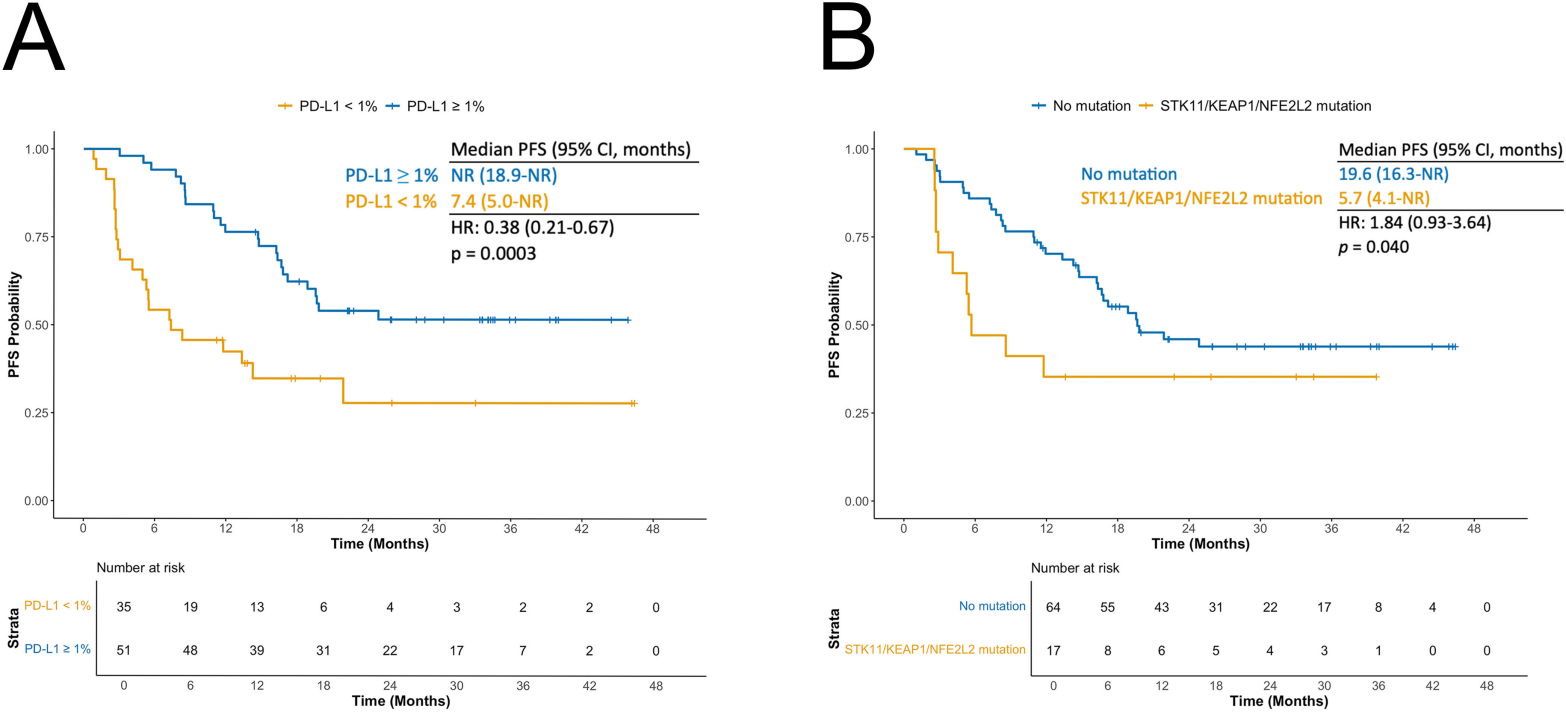
**(A)** Kaplan-Meier curves for progression-free survival (PFS) according to PD-L1 status. **(B)** Kaplan-Meier curves for PFS according to *STK11*/*KEAP1*/*NFE2L2* mutation status.

Patients with PD-L1 tumor expression ≥ 1% had improved PFS compared to those with PD-L1 < 1% (HR: 0.38; 95% CI: 0.21–0.67; *p* = 0.0003; **Figure 4A**). When combining bTMB and PD-L1 status, the longest PFS was observed in patients with both PD-L1 ≥ 1% and high bTMB, using either the 8.5 mut/Mb (**Supplementary Figure 5A**) or 6.6 mut/Mb (**Supplementary Figure 5B**) cut-offs. Compared to the reference group (PD-L1 < 1% and low bTMB), those with PD-L1 ≥ 1% and bTMB ≥ 8.5 mut/Mb had a significantly reduced risk of progression or death (HR: 0.29; 95% CI: 0.13–0.65; *p* = 0.001). The association was even stronger when using the 6.6 mut/Mb cut-off value (HR: 0.22; 95% CI: 0.10-0.50, *p* < 0.001).

### 3.6 Association between genomic alterations in blood and PFS

In univariable analysis, the presence of mutations in *STK11*, *KEAP1*, or *NFE2L2* in plasma was associated with shorter PFS (HR 1.84, 95% CI 0.93–3.64; p = 0.040). Median PFS was 5.7 months (95% CI 4.1–NR) in patients with ≥1 of these mutations versus 19.6 months (95% CI 16.3–NR) in wild-type patients (**Figure 4B**). Combining *STK11/KEAP1/NFE2L2* status with bTMB identified a particularly favorable cohort: patients with wild-type *STK11/KEAP1/NFE2L2* and high bTMB (>8.5 mut/Mb) had an HR of 0.37 (95% CI 0.14–1.03; p = 0.029) compared with those with *STK11/KEAP1/NFE2L2* alterations and low bTMB. Associations with PFS for other ctDNA-detected alterations present in ≥10 patients are shown in **Supplementary Figure 6**. *KRAS* mutations were linked to longer PFS (HR 0.52, 95% CI 0.21–1.33; p = 0.087). Prognosis improved further in *KRAS*-mutated patients after excluding those with *STK11* or *KEAP1* co-mutations (HR 0.35, 95% CI 0.11–1.12; p = 0.034).

### 3.7 Multivariable analysis of factors associated with PFS

**Figure 5:**
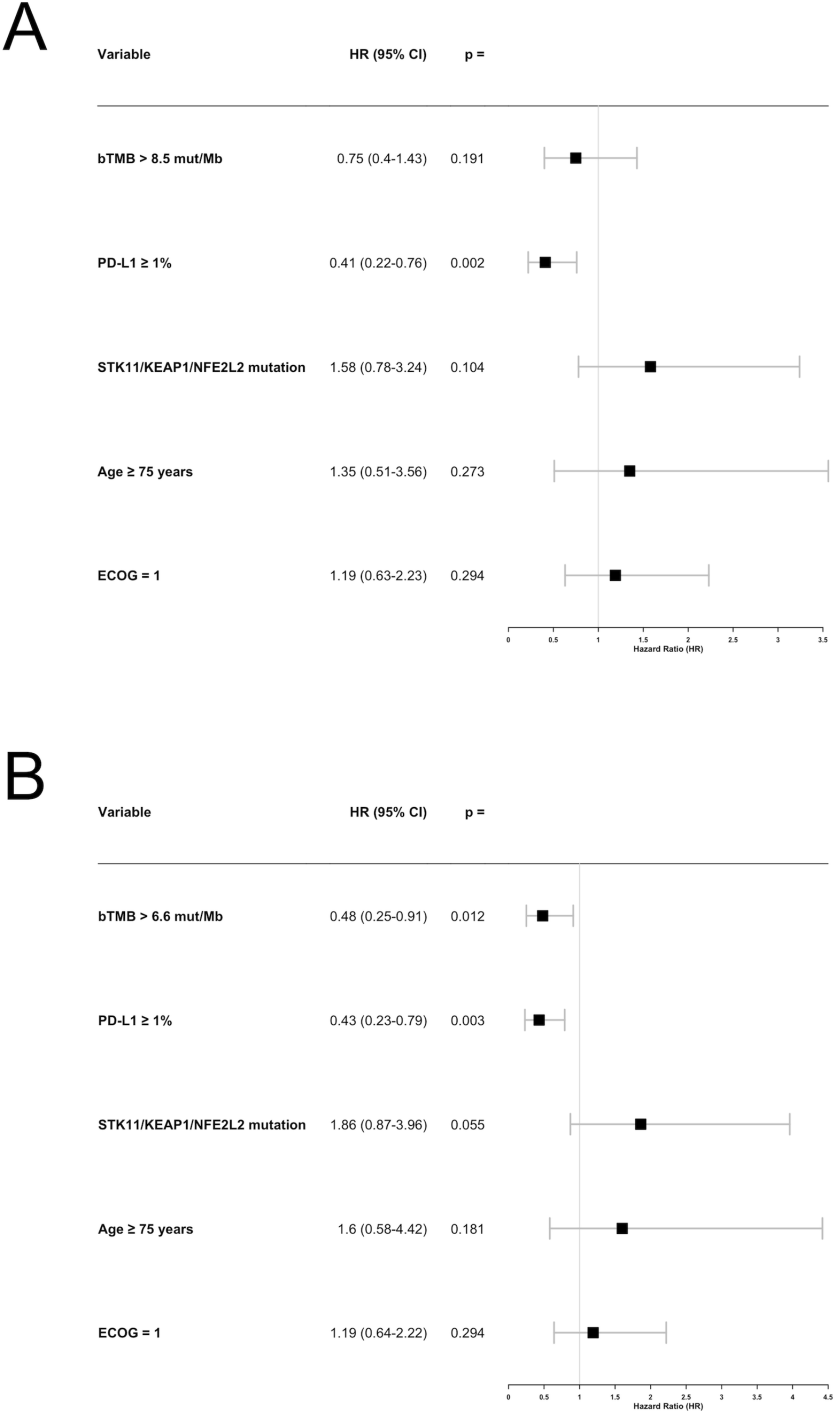
Forest plot for the multivariable analysis of factors associated with progression-free survival (PFS). **(A)** Using the blood tumor mutational burden (bTMB) cut-off of 8.5 mutations per megabase (mut/Mb). **(B)** Using the bTMB cut-off of 6.6 mut/Mb. bTMB, blood tumor mutational burden; mut/Mb, mutations per megabase.

Since high bTMB was associated with longer PFS in univariable analyses using both the prespecified 8.5 mut/Mb cut-off and the median value of 6.6 mut/Mb, we performed two separate multivariable analyses for these cut-offs. In the 8.5 mut/Mb model, only PD-L1 expression ≥ 1% was significantly associated with longer PFS (HR: 0.41; 95% CI: 0.22–0.76; p = 0.002), while *STK11*/*KEAP1*/*NFE2L2* mutations showed a trend toward shorter PFS (HR: 1.58; 95% CI: 0.78–3.24; p = 0.104; **Figure 5A**). In the 6.6 mut/Mb model, high bTMB (HR: 0.48; 95% CI: 0.25–0.91; p = 0.012), PD-L1 ≥ 1% (HR: 0.43; 95% CI: 0.23–0.79; p = 0.003), and *STK11*/*KEAP1*/*NFE2L2* mutations (HR: 1.86; 95% CI: 0.87–3.96; p = 0.055) were all significantly associated with PFS, with high bTMB and PD-L1 ≥ 1% linked to longer PFS and *STK11/KEAP1/NFE2L2* mutations linked to shorter PFS (**Figure 5B**).

## 4 Discussion

In this prospective cohort study of patients with unresectable stage III NSCLC treated with CRT and durvalumab, high bTMB and PD-L1 ≥ 1% were associated with longer PFS, while ctDNA-detected mutations in *STK11*, *KEAP1*, or *NFE2L2* were linked to shorter PFS. These findings provide insight into treatment response and resistance and reveal potential biomarkers to guide clinical decision-making in locally advanced NSCLC.

While TMB is a known predictor of immunotherapy benefit in stage IV NSCLC, its role in locally-advanced disease treated with multimodal therapy remains less established. Recently, retrospective analyses have reported high tTMB to be associated with longer disease control after CRT and consolidative durvalumab (25, 26). However, in locally advanced NSCLC, obtaining sufficient tumor tissue for routine diagnostics can be challenging, often leaving too little material for tTMB and additional biomarker analysis (9, 36). In our trial, only 36 of 81 patients had baseline tumor tissue samples with enough tumor content for tTMB determination. ctDNA-based genomic profiling and bTMB assessment offer a practical alternative when tissue is limited, with several advantages: less invasiveness, reduced susceptibility to intra- and intertumoral heterogeneity, and greater feasibility for repeated measurements throughout treatment for dynamic bTMB monitoring (13, 28).

TMB may influence responses to CRT and durvalumab through multiple mechanisms. High TMB is a predictor of immunotherapy benefit. While its predictive value appears diminished when immunotherapy is paired with chemotherapy (37), this might not apply when combined with radiotherapy (25, 26). Tumors with high TMB offer a more immunogenic tumor microenvironment with more tumor neoantigens and increased CD8-positive and PD-1-positive T-cell infiltration, which may increase the vulnerability of tumor cells to the immune-related effects of radiotherapy (21, 24). Additionally, high TMB correlates with alterations in DNA damage response and repair (DDR) genes, which play key roles in radiation repair. In theory, pathogenic mutations in these genes could further increase radiosensitivity (38, 39).

There is currently no consensus on the optimal threshold to define high versus low TMB (11). In our study, both the 6.6 mut/Mb (median) and the protocol-prespecified 8.5 mut/Mb cut-offs were significantly associated with PFS in univariable analyses. However, only when using the 6.6 mut/Mb cut-off value, did the association remain significant in the multivariable analysis. Applying higher cut-off values yielded no significant association between high bTMB and longer PFS, possibly due to the small number of patients classified as high bTMB and limited statistical power. While the FDA approved pembrolizumab for solid tumors with high TMB using a 10 mut/Mb cut-off (40), some trials suggest higher thresholds, in the 80th-90th percentiles, to better predict immunotherapy efficacy (41, 42). Conversely, a meta-analysis by Meng et al. indicated that lower cut-offs may more effectively identify patients likely to benefit from immunotherapy (11). Ultimately, the optimal threshold likely depends on tumor type, disease stage, methodology, and assay, making it difficult to define a universal standard (13).

Consistent with findings from the PACIFIC and PACIFIC-R studies, we found that patients with PD-L1 tumor expression ≥ 1% had better PFS after CRT and durvalumab compared to PD-L1-negative patients (5, 43). In our cohort, PD-L1, treated as a dichotomous variable with a 1% cut-off value, was the biomarker most strongly associated with PFS, both in univariable and multivariable analyses, reinforcing its clinical relevance in this setting. However, some trials categorizing PD-L1 expression into multiple levels have reported similar outcomes in PD-L1 negative and PD-L1 low (1-49%) disease (26, 44), suggesting that PD-L1 might be better evaluated as a continuous variable and in combination with other biomarkers. Our data support bTMB and PD-L1 as independent markers with the longest PFS observed in patients with both high bTMB and PD-L1 ≥ 1%. While exploratory rather than practice-changing, these results support a multi-biomarker approach integrating PD-L1, TMB, and additional tumor features to refine prognosis and guide treatment selection for this patient group.

Our data indicate that pathogenic mutations in *STK11*, *KEAP1*, and *NFE2L2*, as detected by ctDNA analysis, are associated with inferior PFS in patients undergoing CRT and durvalumab. These mutations have been linked to increased resistance to radiotherapy (24, 29, 30). Increasing evidence also supports that tumors with *STK11* or *KEAP1* mutations are less responsive to chemotherapy and PD-L1-targeted immunotherapy, suggesting their role as negative prognostic biomarkers (45, 46). If our findings and a median PFS of six months reflect the expected benefit of CRT and durvalumab in this subgroup, risk-adaptive strategies could be warranted. Subgroup analyses from POSEIDON and CheckMate 227 suggest that adding a CTLA-4 inhibitor may improve outcomes in metastatic NSCLC with *STK11* and *KEAP1* mutations (47, 48). However, given the already intensive combination of CRT and durvalumab, further escalation of treatment for a minor improvement in outcome should be considered with caution. Novel therapeutics targeting *STK11*- and *KEAP1*/*NRF2* pathways are being investigated and could play a role in the future (45). Importantly, not all *STK11/KEAP1/NFE2L2*-mutations are equally deleterious. Mutation subtype, clonality, the broader genomic landscape and co-mutations (particularly *KRAS)* should be factored in when assessing the clinical impact of these alterations.

Although high bTMB was associated with longer PFS, our multivariable analyses suggest it may not be sufficiently robust as a stand-alone biomarker in unresectable, locally-advanced NSCLC. Combining bTMB with additional molecular markers, such as pathogenic gene alterations, could better capture the tumor’s molecular characteristics and improve outcome prediction (8, 9). In our trial, three patients with bTMB > 20 mut/Mb experienced disease progression within 12 months, all of whom had deleterious mutations in *STK11*, *KEAP1*, or *NFE2L2*. Furthermore, the association between high bTMB and longer PFS was strengthened when patients with these mutations were excluded. A similar finding was reported by Shaverdian et al., where high tTMB predicted improved locoregional control following post-operative radiotherapy, primarily in NSCLC patients without mutations in genes associated with radioresistance (24). In our study, the combination of high bTMB and *STK11*/*KEAP1*/*NFE2L2* wild-type status identified a subgroup with a particularly favorable prognosis. If validated in future trials, this cohort may be considered for treatment de-intensification, such as reduced duration of durvalumab therapy.

A combinatorial strategy could incorporate not only bTMB, PD-L1 status, and pathogenic mutations in key genes, but potentially also factors such as cytokines, immune cell composition, and tumor microenvironment features (13). A multi-biomarker model may provide a stronger foundation for personalized treatment. However, for such an approach to be clinically applicable, it must be practical, time-efficient, and cost-effective. Most importantly, further prospective validation is needed before implementing these biomarker-guided strategies in routine clinical practice.

Some limitations of this study should be acknowledged. Its exploratory nature is reflected in the small size of certain genetic subgroups and the use of a significance level of 0.10. Survival data are still immature, and it remains to be seen whether differences in PFS between biomarker-related subgroups will translate into OS differences. As all patients received the same treatment, it is also difficult to determine whether the investigated biomarkers are predictive or merely prognostic. For comparisons of bTMB vs. tTMB, and plasma vs. tissue-based mutation detection, different assays (targeted panel vs. WES) and reference genomes (GRCh37 vs. GRCh38) were used, which limits strict variant-level matching without liftover/re-validation. Thus, there are potentially several technical reasons in addition to biological reasons for the moderate concordance previously reported (49). Finally, only 36 patients had baseline tissue samples with sufficient tumor content for sequencing, and in some cases, DNA concentrations were below the recommended threshold (10 ng/µl), increasing the uncertainty of the tTMB results.

In conclusion, high bTMB and PD-L1 expression ≥ 1% were associated with longer PFS in patients with stage III NSCLC undergoing CRT and consolidative durvalumab, while ctDNA-detected pathogenic mutations in *STK11*, *KEAP1*, or *NFE2L2* were linked to shorter PFS. Future studies are needed to validate these as complementary biomarkers and to explore personalized treatment strategies, including risk-adapted escalation or de-escalation of therapy.

## Data Availability

The datasets presented in this article are not readily available because they contain information that could compromise participant privacy. Requests to access the datasets should be directed to the corresponding author, Henrik Horndalsveen (henrik.horndalsveen@gmail.com). Summary-level data supporting the conclusions of this article are included in the article and its **Supplementary Material**.
