# Supplementary material for: Blood-based tumor mutational burden as a biomarker in unresectable non-small cell lung cancer treated with chemoradiotherapy and durvalumab
Source: Front Oncol. 2025 Oct 22;15:1681420. doi: 10.3389/fonc.2025.1681420 (PMC12586078; doi:10.3389/fonc.2025.1681420)
Supplement: Supplementary file 1 [file DataSheet1.pdf]

Supplementary Methods

Tissue sampling and DNA extraction

Tumor tissue

Formalin-fixed paraffin-embedded (FFPE) tumor tissue and matching buffy coat samples for germline variant determination were obtained at baseline. HE-stained FFPE tissue sections underwent an initial histopathological review by an experienced pathologist to exclude samples with inadequate tumor content. DNA was extracted from FFPE tissue using the AllPrep DNA/RNA FFPE Kit (Qiagen) on a QiaCube Connect according to the manufacturer's instructions and from buffy coats using the QIAamp DNA Blood Mini Kit (Qiagen) with some protocol modifications due to blood collection in Cell-Free DNA (cfDNA) BCT tubes (Streck). DNA was quantified with Qubit dsDNA BR Assay on a Qubit 4 fluorometer (ThermoFisher Scientific). DNA quality was assessed by the OD ratios A_{260}/A_{280} and A_{260}/A_{230}, using Nanodrop One (ThermoFisher Scientific), and fragment length, via Genomic DNA ScreenTape Analysis (Agilent). Only FFPE-derived samples with a DNA concentration > 3 ng/µl and matching buffy coats were submitted for sequencing.

Plasma samples

Three 10 ml cfDNA BCT tubes (Streck) of peripheral blood were collected from each patient at screening. Plasma was separated by two-step centrifugation: first at 1600g for 10 minutes, then at 16000g for 10 minutes, and conserved at -80°C. cfDNA was extracted from 8 ml plasma using the Mag-Bind cfDNA kit (Omega Bio Tek, M3298-02) and an automated pipeline (Opentrons OT-2, KingFisher Flex) following the manufacturer's instructions.

Library preparation, enrichment, and sequencing

Tumor tissue

DNA library preparation and exome capturing from the tumor/buffy coat pairs were done at the OUH Genomics Core Facility using Twist Biosciences Library Preparation Kit (Illumina) and Twist Human Comprehensive Exome Enrichment Kit (Illumina). Final libraries were sequenced paired-end 2 x 150 bp on the NovaSeq6000 system (Illumina) with an average coverage of 150x for tumor and 50x for buffy coat.

Plasma samples

DNA library preparation followed established protocols (1). Extracted cfDNA underwent dA-tailing and subsequent adaptor ligation. DNA concentration was assessed with digital droplet PCR. A clean-up step was performed using magnetic beads, followed by indexing PCR before a final clean-up. DNA libraries were evaluated using the 4150 Agilent Tapestation system (D1000 ScreenTape, Agilent). Targeted genomic regions were enriched through a hybridization-based method using TACS (target capture sequences) tailored to capture designated loci in the genes of interest. The NeoThetis Pan Cancer Plus assay (MEDICOVER Genetics) was used to identify single nucleotide variants (SNVs), small insertions and deletions (indels), copy number amplifications (CNAs) and structural rearrangements (Supplementary Table 1). Captured DNA fragments were eluted by heating and amplified using outer-bound adaptor primers. Finally, the enriched cfDNA samples were pooled

equimolarly and sequenced on a Novaseq 6000 platform (Illumina). All sequenced cfDNA libraries passed quality control analysis.

Variant calling, annotation and TMB calculation

Tumor tissue

Poor-quality reads and terminal adaptor sequences were removed before aligning raw sequencing reads from tumor tissue and matched buffy coat samples to the human reference genome GRCh38, using the Burrows–Wheeler Aligner (BWA_MEM2). Somatic variants were identified with GATK Mutect2 (v4.2.6.1) and Strelka (v2.9.10), using matched buffy coat samples to filter germline variants and retain tumor-specific mutations. Annotation of somatic variants was done using the Personal Cancer Genome Reporter (PCGR) software package (2). A variant allele frequency (VAF) threshold ≥ 5 % and a tumor sample read depth threshold ≥ 100x were applied to exclude low-confidence variants before TMB calculation. TMB was calculated as the number of non-synonymous SNVs and indels per megabase (mut/Mb) of targeted exome.

Plasma samples

Sequenced reads were demultiplexed using bcl-convert (v4.2) with poor-quality reads and adaptor sequences removed from the paired-end read DNA fragments. Utilizing the Burrows-Wheeler alignment algorithm, the remaining sequences were aligned to the human reference genome GRCh37 (3). Duplicate reads were detected, sorted by unique adaptor families, and processed to generate consensus reads per family. To further refine the set of positive variant calls, a statistical error correction model (at base-pair resolution), followed by a filtering bioinformatics pipeline was applied. Variants with VAF < 0.25%, synonymous variants, low-confidence variants, and variants with >1% population frequency (based on the gnomAD database) were excluded. For bTMB estimation, only targeted genomic regions with a minimum read depth of 1000x were evaluated. SNVs and small indels that passed all filtering steps, were included. The bTMB scoring system achieved a high concordance correlation coefficient between reference and estimated bTMB scores (r=0.93; 95% CI:0.88-0.95).

1. Kyrochristos ID, Glantzounis GK, Goussia A, Eliades A, Achilleos A, Tsangaras K, Hadjidemetriou I, Elpidorou M, Ioannides M, Koumbaris G, et al. Proof-of-Concept Pilot Study on Comprehensive Spatiotemporal Intra-Patient Heterogeneity for Colorectal Cancer With Liver Metastasis. *Front Oncol* (2022) 12:855463. doi: 10.3389/fonc.2022.855463
2. Nakken S, Fournous G, Vodák D, Aasheim LB, Myklebost O, Hovig E. Personal Cancer Genome Reporter: variant interpretation report for precision oncology. *Bioinformatics* (2018) 34:1778–1780. doi: 10.1093/BIOINFORMATICS/BTX817
3. Li H. Aligning sequence reads, clone sequences and assembly contigs with BWA-MEM. *eprint arXiv* (2013)
