# Supplementary material for: Blood-based tumor mutational burden as a biomarker in unresectable non-small cell lung cancer treated with chemoradiotherapy and durvalumab
Source: Front Oncol. 2025 Oct 22;15:1681420. doi: 10.3389/fonc.2025.1681420 (PMC12586078; doi:10.3389/fonc.2025.1681420)

Supplementary Figure 1

Boxplot showing the distribution of blood-based tumor mutational burden (bTMB) according to PD-L1 tumor expression status.

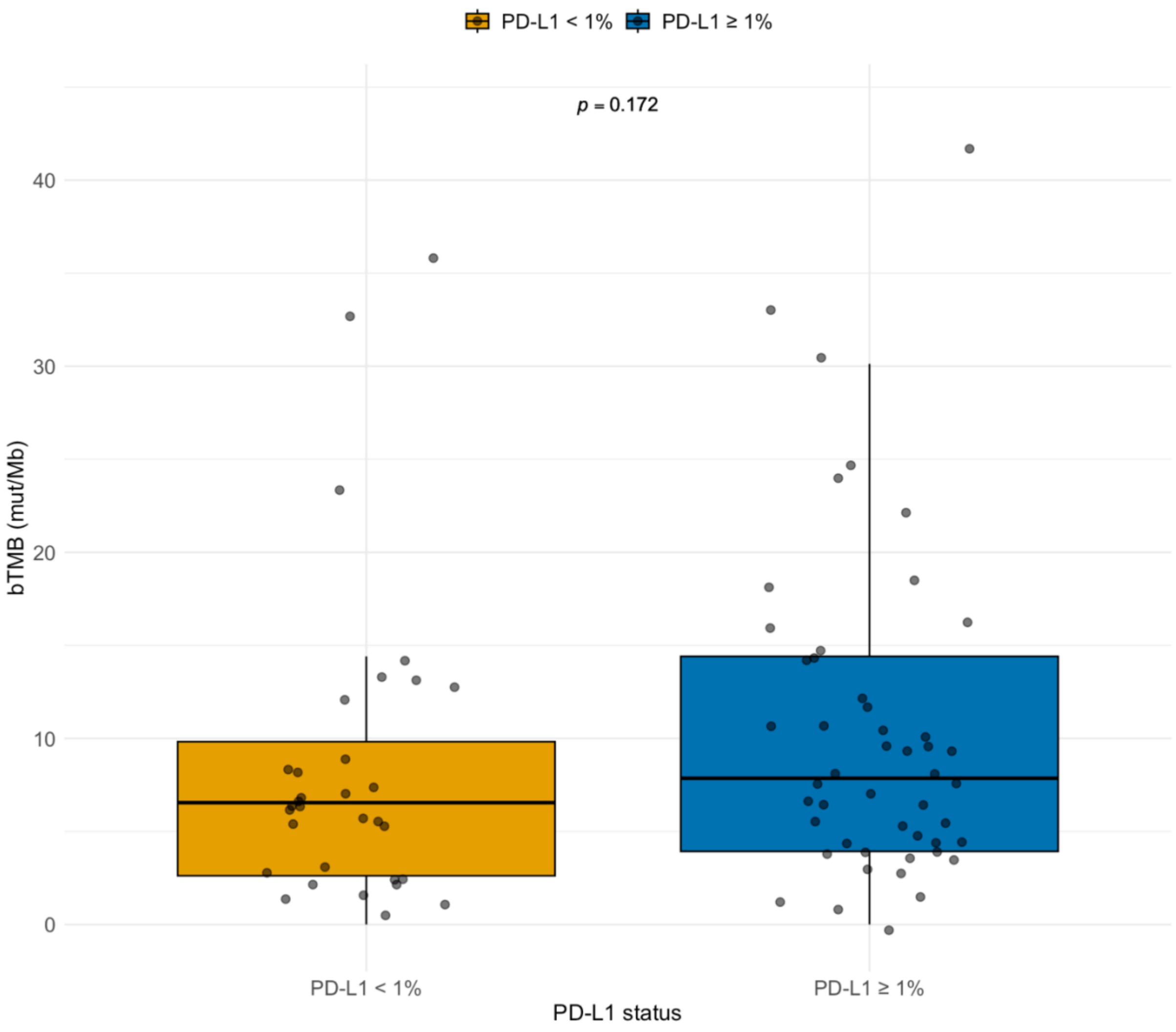

Supplement: Supplementary file 2 [file DataSheet2.pdf]
