# Supplementary material for: Blood-based tumor mutational burden as a biomarker in unresectable non-small cell lung cancer treated with chemoradiotherapy and durvalumab
Source: Front Oncol. 2025 Oct 22;15:1681420. doi: 10.3389/fonc.2025.1681420 (PMC12586078; doi:10.3389/fonc.2025.1681420)

Supplementary Figure 2

Heatmap comparing detected single-nucleotide variants (SNVs) and indels between blood and tumor tissue for the 36 patients with tumor tissue available for whole-exome sequencing (WES). Rows show genes and columns show patients. Each gene–patient cell is classified as No mutations detected**,** Tissue only**,** Blood only**,** or Both tissue and blood**.**

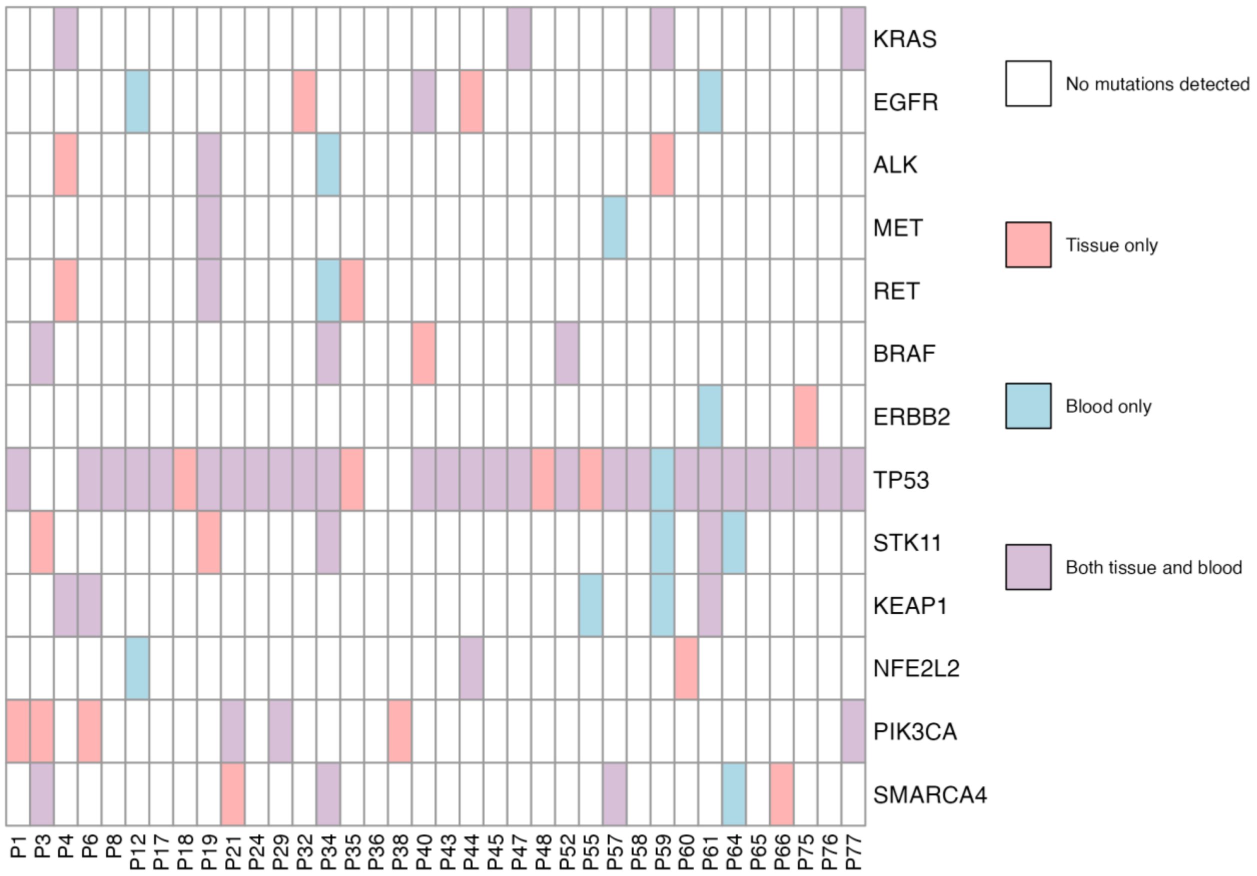

Supplement: Supplementary file 3 [file DataSheet3.pdf]
