# Supplementary material for: Blood-based tumor mutational burden as a biomarker in unresectable non-small cell lung cancer treated with chemoradiotherapy and durvalumab
Source: Front Oncol. 2025 Oct 22;15:1681420. doi: 10.3389/fonc.2025.1681420 (PMC12586078; doi:10.3389/fonc.2025.1681420)

Supplementary Figure 3

Forest plot for univariable analysis of the association between baseline clinical characteristics and progression-free survival (PFS).
CRT: chemoradiotherapy; Durva: durvalumab; PFS: progression-free survival.

Subgroup	N	Hazard Ratio (95% CIs) for PFS	P-value
**Sex**			
Female	34		
Male	52	HR: 1.63 (0.88-3.02)	p = 0.06
**Age**			
< 75 years	79		
≥ 75 years	7	HR: 2.02 (0.80-5.13)	p = 0.07
**Smoking**			
Never	4		
Previous	56	HR: 1.42 (0.34-5.90)	p = 0.32
Current	26	HR: 1.13 (0.25-5.04)	p = 0.44
**ECOG**			
0	34		
1	52	HR: 1.29 (0.71-2.34)	p = 0.21
**Histology**			
Adenocarcinoma	31		
Squamous cell carcinoma	49	HR: 1.05 (0.57-1.91)	p = 0.44
Other	6	HR: 0.59 (0.14-2.53)	p = 0.24
**Stage**			
IIIA	38		
IIIB	39	HR: 1.02 (0.56-1.86)	p = 0.47
IIIC	8	HR: 0.70 (0.21-2.34)	p = 0.28
**Time CRT - Durva**			
< 28 days	57		
≥ 28 days	29	HR: 0.91 (0.49-1.69)	p = 0.38

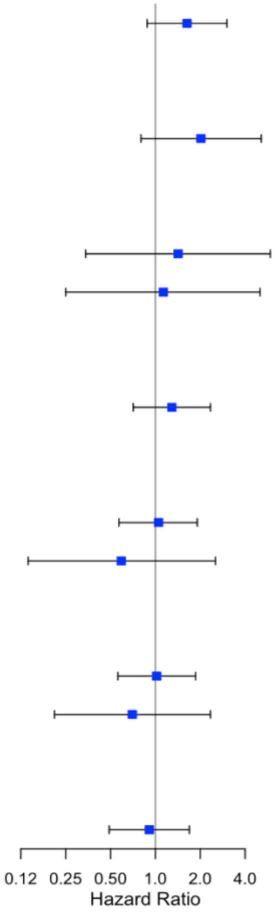

Supplement: Supplementary file 4 [file DataSheet4.pdf]
