# Supplementary material for: Blood-based tumor mutational burden as a biomarker in unresectable non-small cell lung cancer treated with chemoradiotherapy and durvalumab
Source: Front Oncol. 2025 Oct 22;15:1681420. doi: 10.3389/fonc.2025.1681420 (PMC12586078; doi:10.3389/fonc.2025.1681420)
Supplement: Supplementary file 5 [file DataSheet5.pdf]

Supplementary Figure 4

Association between blood tumor mutational burden (bTMB) and progression-free-survival (PFS) based on additional exploratory cut-offs to distinguish between high and low bTMB:
(A) bTMB </> 10 mutations per megabase (mut/Mb).
(B) bTMB </> 16 mut/Mb
(C) bTMB </> 20 mut/Mb.

A

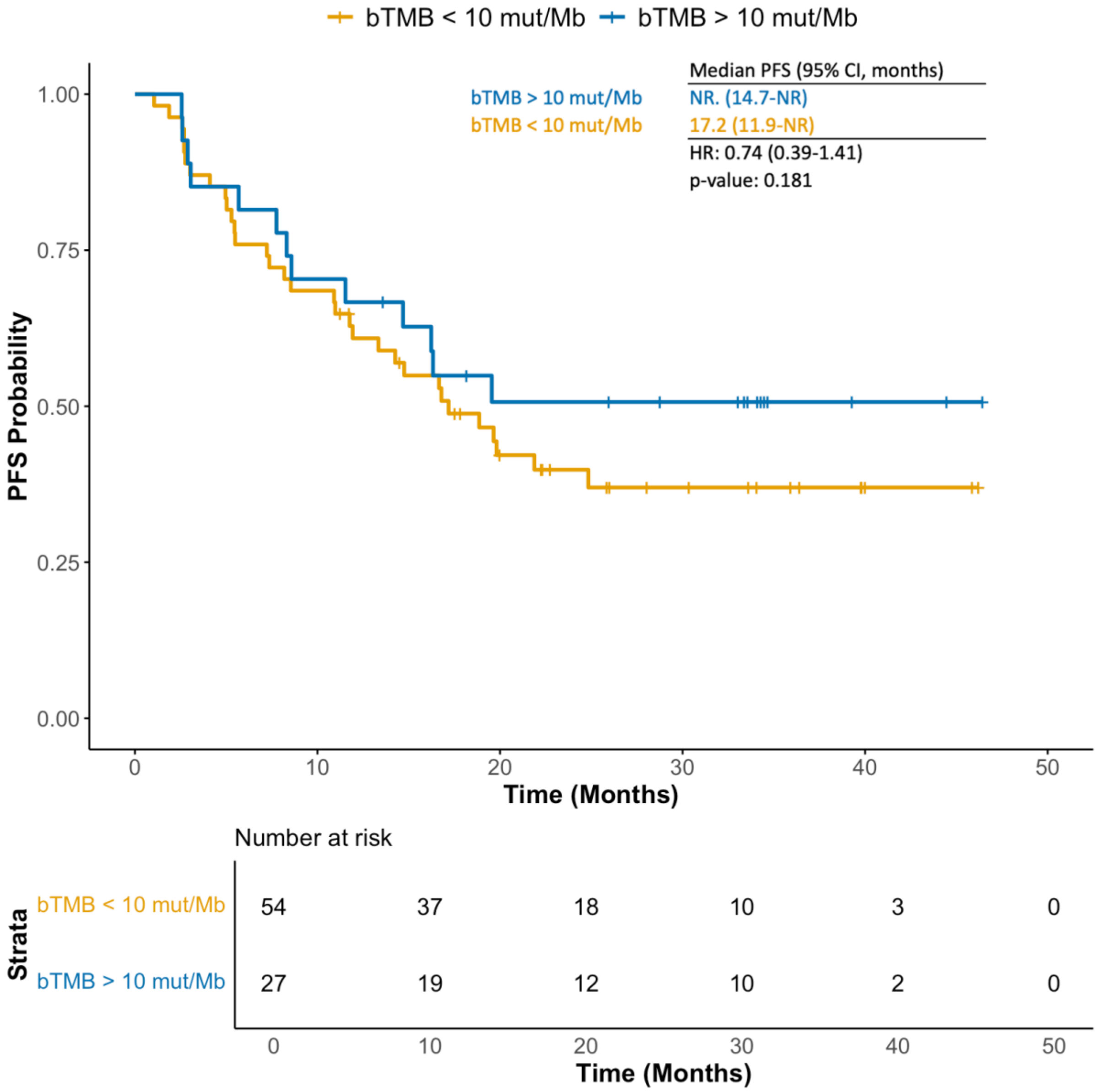

B

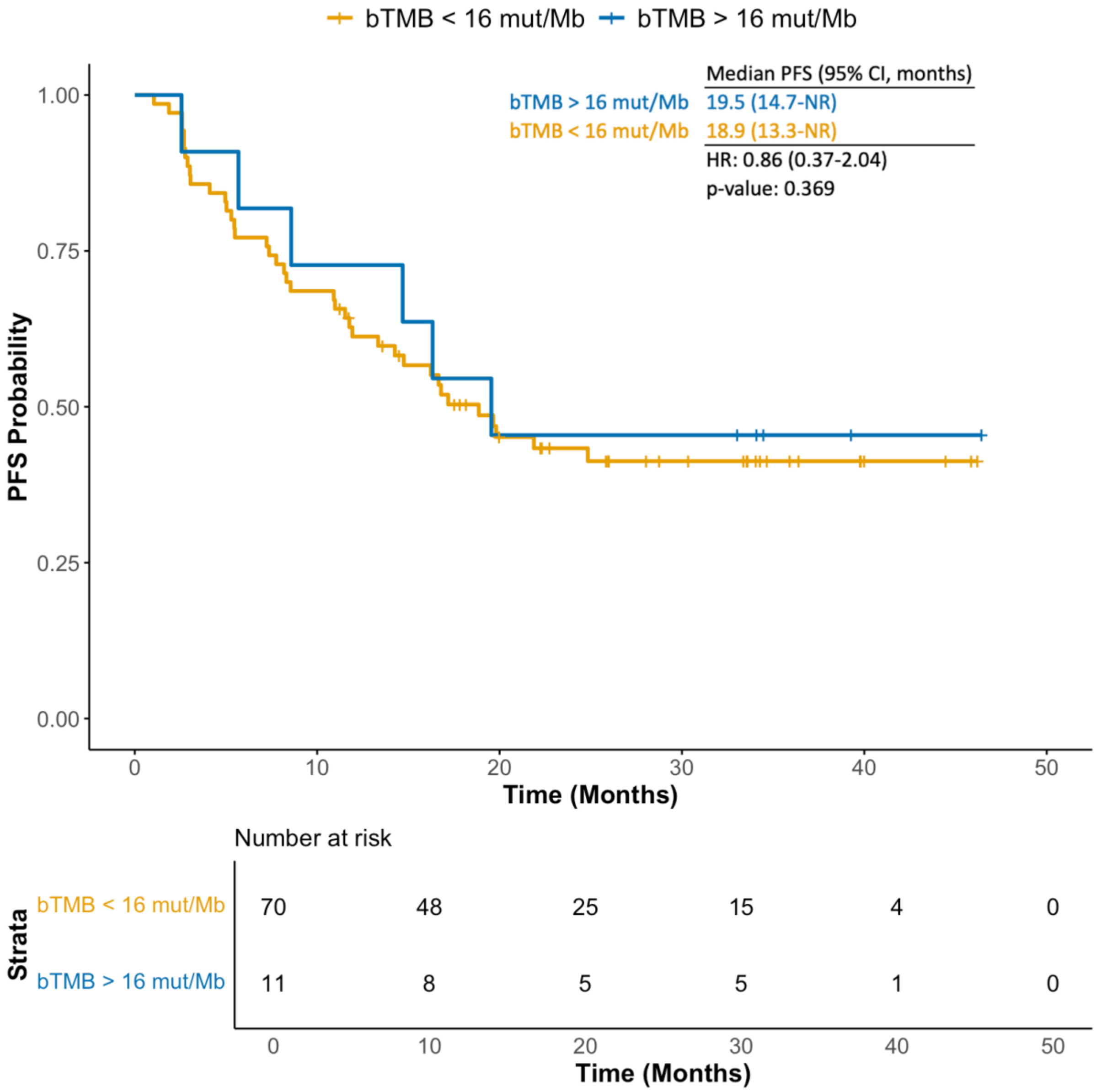
bTMB < 16 mut/Mb
bTMB > 16 mut/Mb
Median PFS (95% CI, months)
bTMB > 16 mut/Mb 19.5 (14.7-NR)
bTMB < 16 mut/Mb 18.9 (13.3-NR)
HR: 0.86 (0.37-2.04)
p-value: 0.369
PFS Probability
1.00
0.75
0.50
0.25
0.00
0
10
20
30
40
50
Time (Months)
Number at risk
Strata
bTMB < 16 mut/Mb 70 48 25 15 4 0
bTMB > 16 mut/Mb 11 8 5 5 1 0
Time (Months)

C

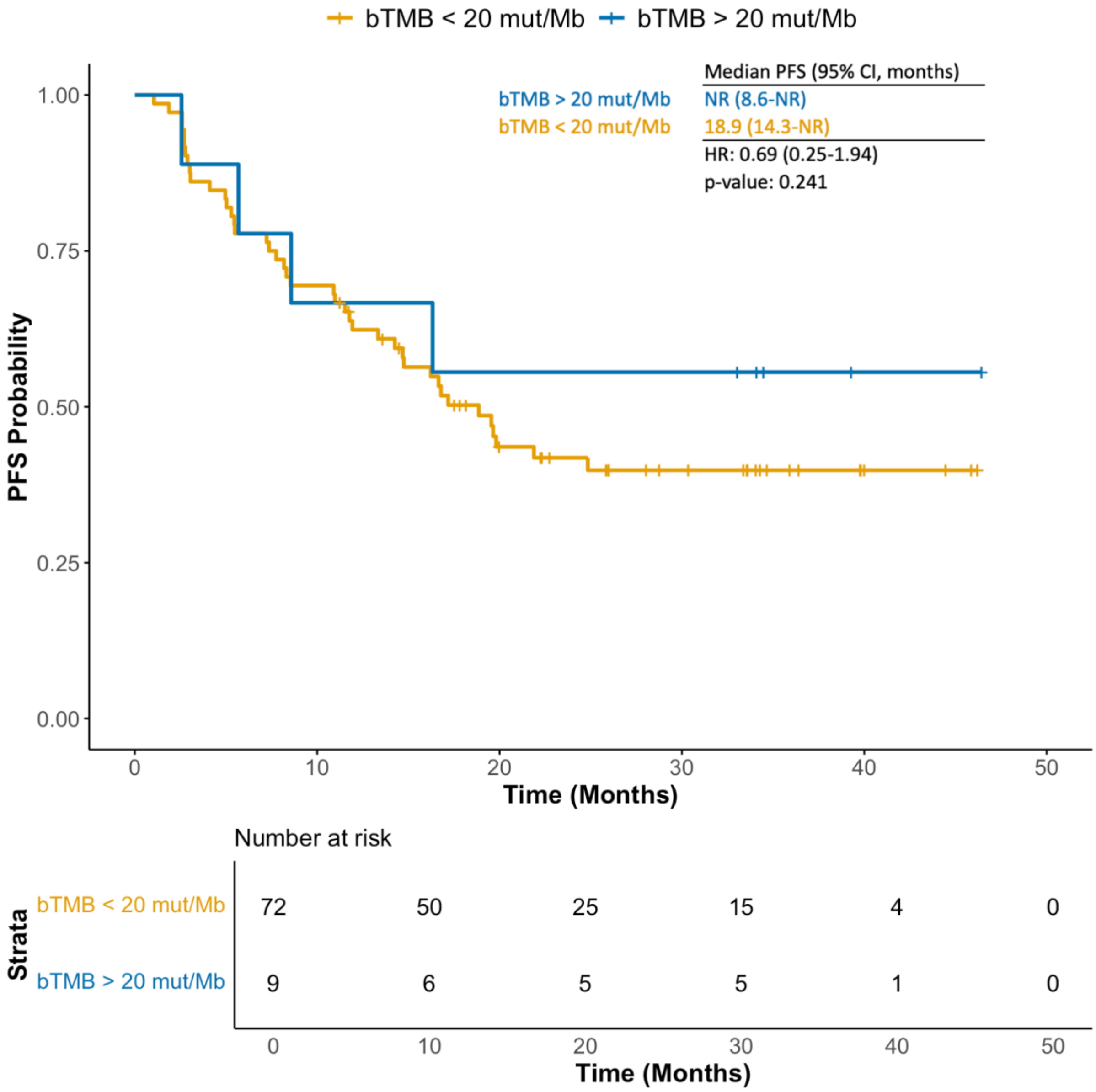
bTMB < 20 mut/Mb
bTMB > 20 mut/Mb
Median PFS (95% CI, months)
bTMB > 20 mut/Mb
NR (8.6-NR)
bTMB < 20 mut/Mb
18.9 (14.3-NR)
HR: 0.69 (0.25-1.94)
p-value: 0.241
PFS Probability
1.00
0.75
0.50
0.25
0.00
0
10
20
30
40
50
Time (Months)
Number at risk
Strata
bTMB < 20 mut/Mb
72
50
25
15
4
0
bTMB > 20 mut/Mb
9
6
5
5
1
0
0
10
20
30
40
50
Time (Months)
