# Supplementary material for: Blood-based tumor mutational burden as a biomarker in unresectable non-small cell lung cancer treated with chemoradiotherapy and durvalumab
Source: Front Oncol. 2025 Oct 22;15:1681420. doi: 10.3389/fonc.2025.1681420 (PMC12586078; doi:10.3389/fonc.2025.1681420)
Supplement: Supplementary file 6 [file DataSheet6.pdf]

Supplementary Figure 5

Kaplan-Meier curves for progression-free survival (PFS) based on the combined blood tumor mutational burden (bTMB) and PD-L1 status.
(A) bTMB cut-off = 8.5 mutations per megabase (mut/Mb) + PD-L1 cut off ≥ 1%
(B) bTMB cut-off = 6.6 mut/Mb + PD-L1 cut off ≥ 1%

A

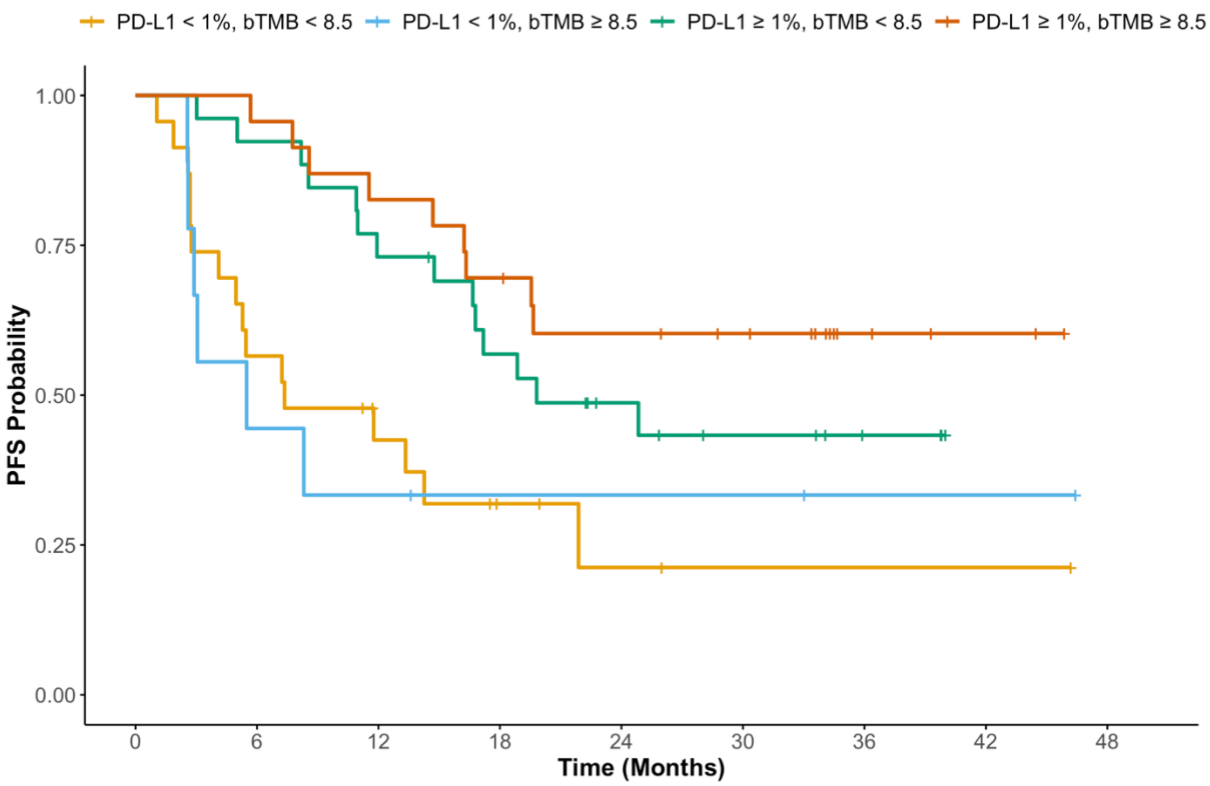

Number at risk

Strata	0	6	12	18	24	30	36	42	48
PD-L1 < 1%, bTMB < 8.5	23	13	8	4	2	1	1	1	0
PD-L1 < 1%, bTMB ≥ 8.5	9	4	3	2	2	2	1	1	0
PD-L1 ≥ 1%, bTMB < 8.5	26	24	19	14	9	6	3	0	0
PD-L1 ≥ 1%, bTMB ≥ 8.5	23	22	19	16	13	11	4	2	0

Time (Months)

B

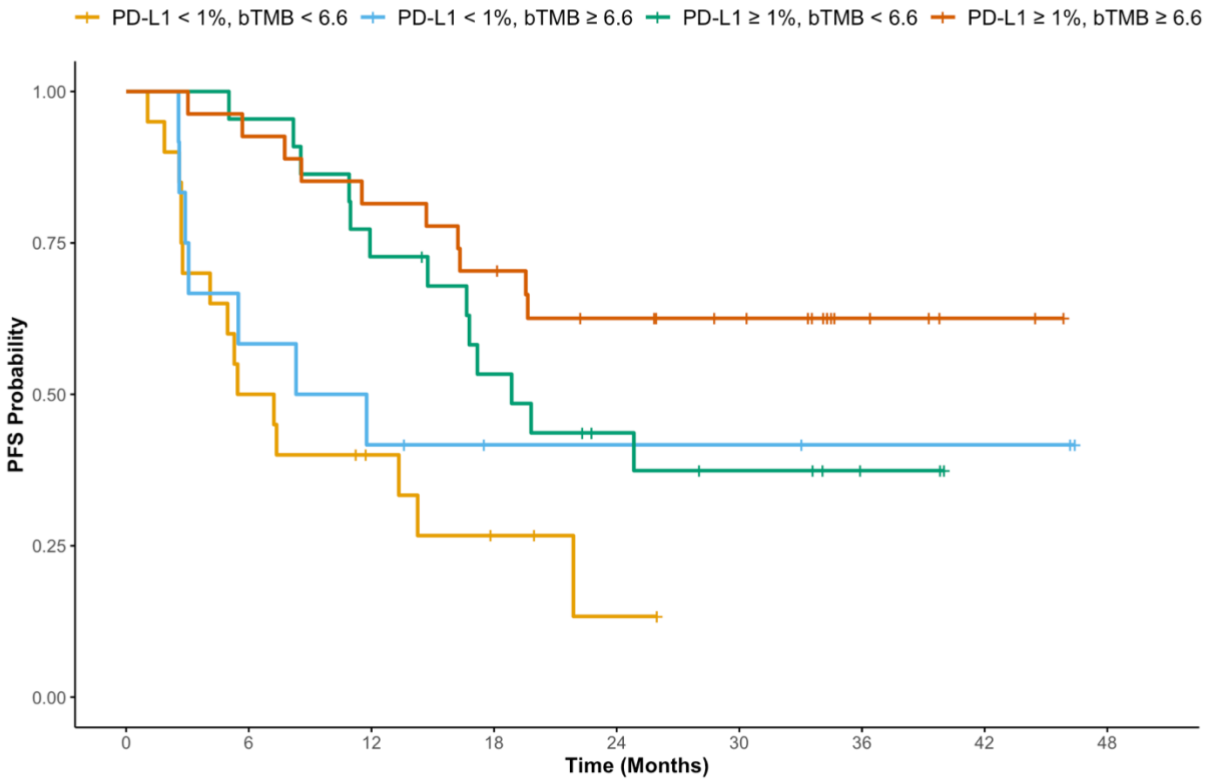

Number at risk

Strata	0	6	12	18	24	30	36	42	48
PD-L1 < 1%, bTMB < 6.6	20	10	6	3	1	0	0	0	0
PD-L1 < 1%, bTMB ≥ 6.6	12	7	5	3	3	3	2	2	0
PD-L1 ≥ 1%, bTMB < 6.6	22	21	16	11	7	5	2	0	0
PD-L1 ≥ 1%, bTMB ≥ 6.6	27	25	22	19	15	12	5	2	0

Time (Months)
