# Supplementary material for: Blood-based tumor mutational burden as a biomarker in unresectable non-small cell lung cancer treated with chemoradiotherapy and durvalumab
Source: Front Oncol. 2025 Oct 22;15:1681420. doi: 10.3389/fonc.2025.1681420 (PMC12586078; doi:10.3389/fonc.2025.1681420)

Supplementary Figure 6

Forest plot displaying hazard ratios (HRs) with 95% confidence intervals (CIs) and *p*-values for PFS. Genomic alterations were detected in blood samples, and only alterations identified in at least 10 patients are presented. Alterations in the *KEAP1* and *NFE2L2* genes are grouped together.

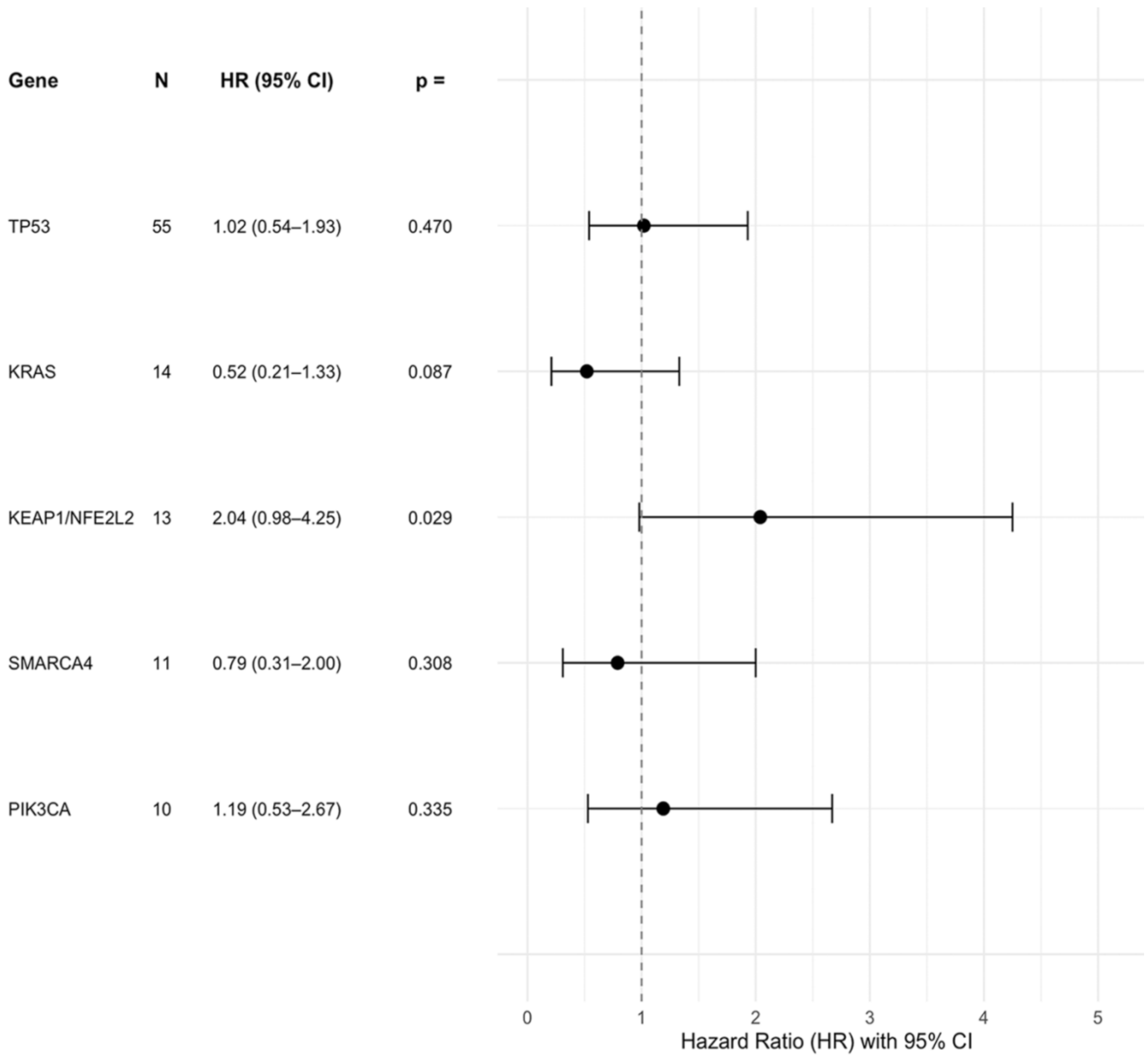

Supplement: Supplementary file 7 [file DataSheet7.pdf]
