# Supplementary figures and images for: Blood-based tumor mutational burden as a biomarker in unresectable non-small cell lung cancer treated with chemoradiotherapy and durvalumab
Source: Front Oncol. 2025 Oct 22;15:1681420. doi: 10.3389/fonc.2025.1681420 (PMC12586078; doi:10.3389/fonc.2025.1681420)

**Supplementary Table 1.** The NeoThetis Pan Cancer Plus Panel.


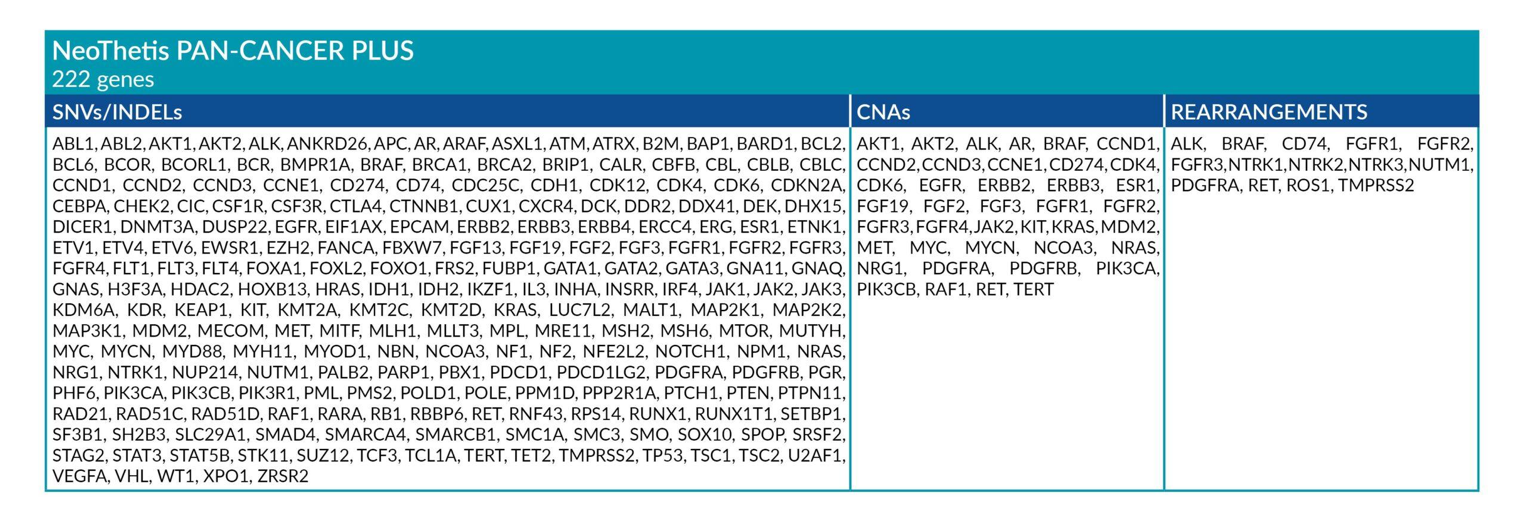

Supplement: Supplementary file 8 [file Table1.docx]
